# Supplementary material for: Association between Different Types of Plant-Based Diets and Dyslipidemia in Middle-Aged and Elderly Chinese Participants
Source: Nutrients. 2023 Jan 2;15(1):230. doi: 10.3390/nu15010230 (PMC9823762; doi:10.3390/nu15010230)
Supplement: Supplementary file 1 [file nutrients-15-00230-s001.zip › nutrients-2076126-supplementary.pdf]

Supplement

Table S1. Partial correlation analysis showing the relationship between dyslipidemia-related indicators and plant-based diet indices

	**PDI**		**hPDI**		**uPDI**		**aPDI**	
	r	*p*	*r*	*p*	*r*	*p*	*r*	*p*
**TC/HDL-C**	-0.003	0.859	-0.038	0.015	0.045	0.004	-0.053	0.001
**LDL-C/HDL-C**	-0.022	0.153	-0.036	0.021	0.018	0.262	-0.034	0.031
**AIP**	-0.034	0.029	-0.050	0.001	0.054	0.001	-0.063	0.000

r is the partial correlation coefficient, controlling for sex, age, ethnicity, BMI, SES, smoking status, alcohol consumption, physical activity, T2DM, and hypertension.

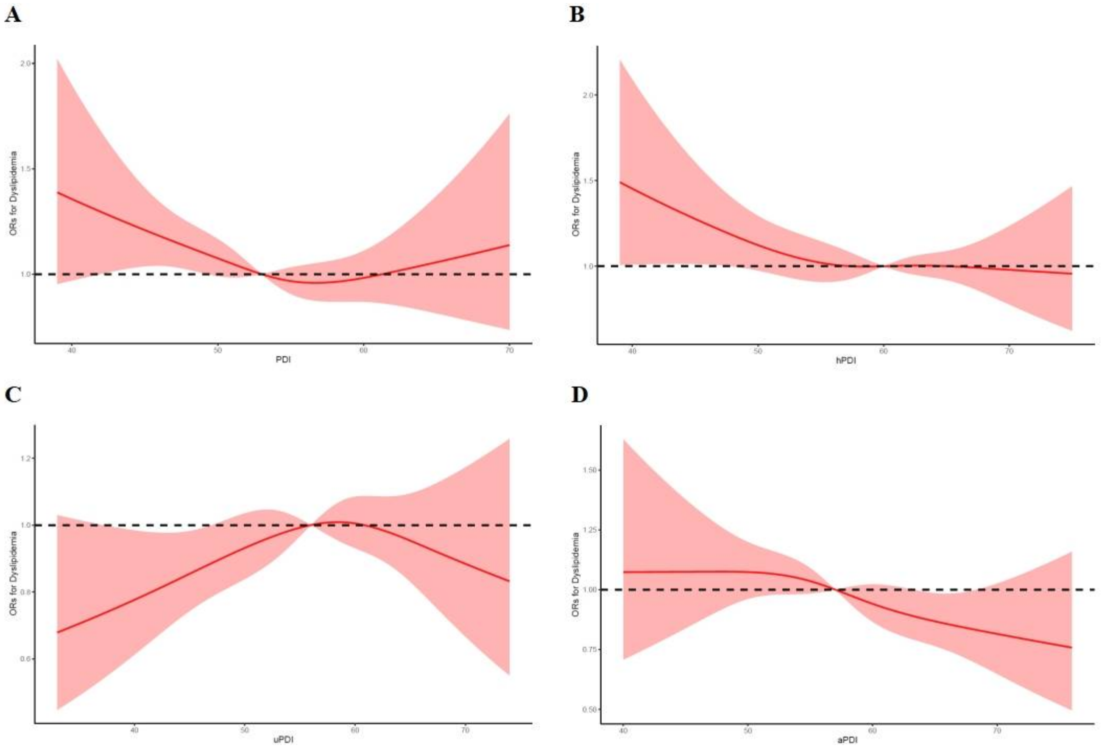

Figure S1. Representation of restricted cubic spline logistic regression models for four plant-based diet indices (PDI, hPDI, uPDI, and aPDI) and risk of dyslipidemia. Red solid line, OR as a function of specific plant-based diet indices adjusted for sex, age, ethnicity, BMI, SES, smoking status, alcohol consumption, physical activity, T2DM, and hypertension; red area,95% CIs. (A) RCS logistic regression models for PDI and ORs of dyslipidemia. (B) RCS logistic regression models for hPDI and ORs of dyslipidemia. (C) RCS logistic regression models for uPDI and ORs of dyslipidemia. (D) RCS logistic regression models for aPDI and ORs of dyslipidemia.

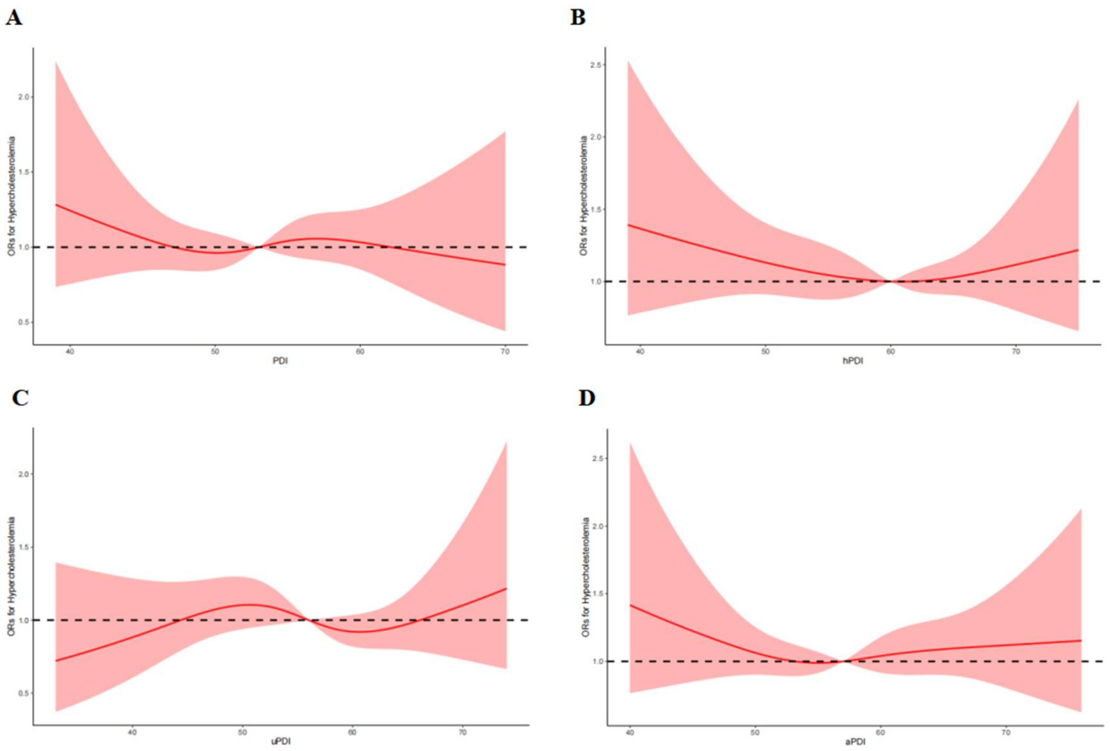

Figure S2. Representation of restricted cubic spline logistic regression models for four plant-based diet indices (PDI, hPDI, uPDI and aPDI) and risk of hypercholesteremia. Red solid line, OR as a function of specific plant-based diet indices adjusted for sex, age, ethnicity, BMI, SES, smoking status, alcohol consumption, physical activity, T2DM, and hypertension; red area,95% CIs. (A) RCS logistic regression models for PDI and ORs of hypercholesteremia. (B) RCS logistic regression models for hPDI and ORs of hypercholesteremia. (C) RCS logistic regression models for uPDI and ORs of hypercholesteremia. (D) RCS logistic regression models for aPDI and ORs of hypercholesteremia.

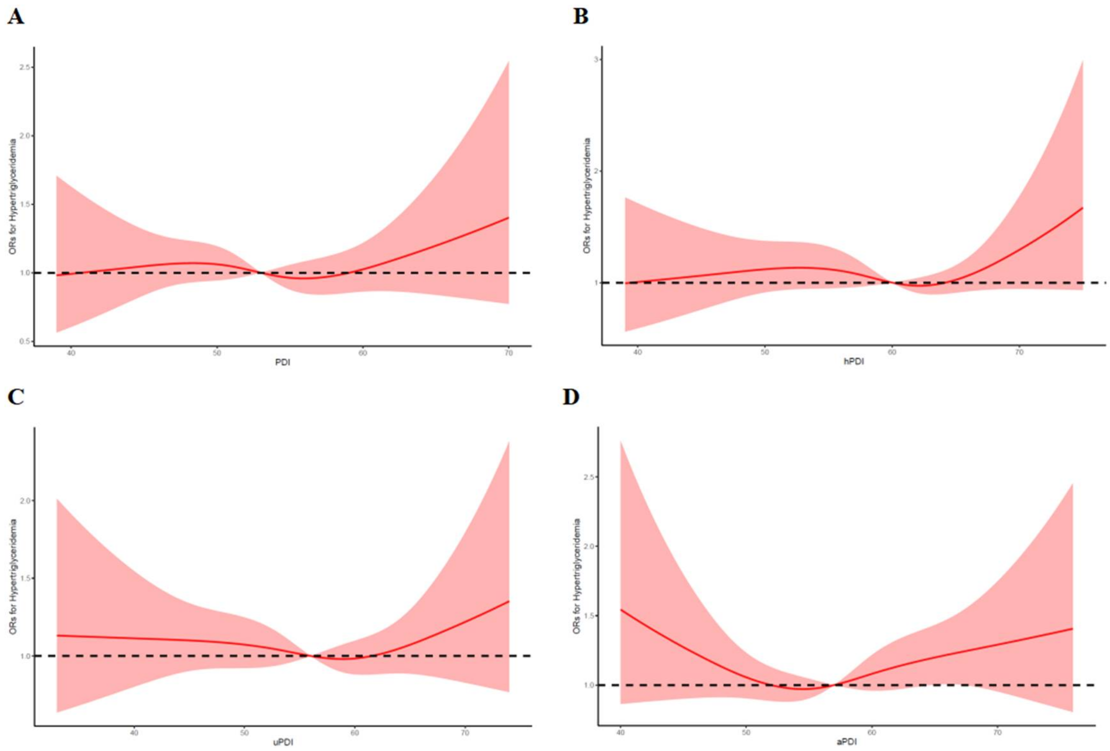

Figure S3. Representation of restricted cubic spline logistic regression models for four plant-based diet indices (PDI, hPDI, uPDI and aPDI) and risk of hypertriglyceridemia. Red solid line, OR as a function of specific plant-based diet indices adjusted for sex, age, ethnicity, BMI, SES, smoking status, alcohol consumption, physical activity, T2DM, and hypertension; red area,95% CIs. (A) RCS logistic regression models for PDI and ORs of hypertriglyceridemia. (B) RCS logistic regression models for hPDI and ORs of hypertriglyceridemia. (C) RCS logistic regression models for uPDI and ORs of hypertriglyceridemia. (D) RCS logistic regression models for aPDI and ORs of hypertriglyceridemia.

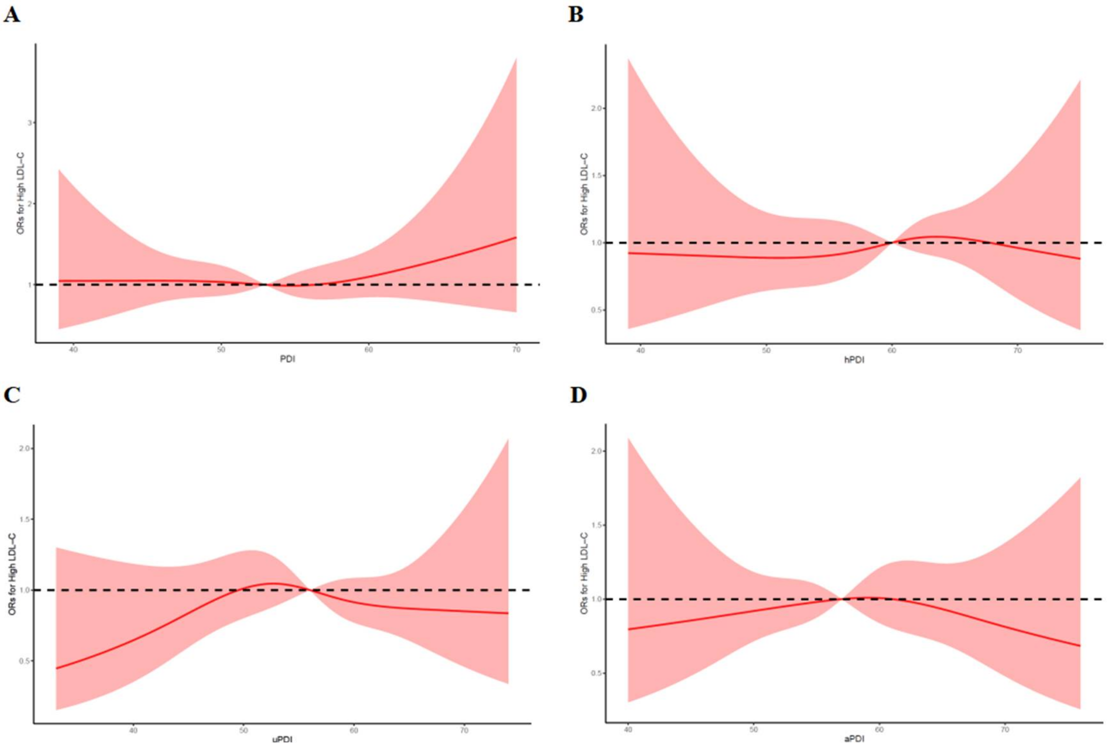

Figure S4. Representation of restricted cubic spline logistic regression models for four plant-based diet indices (PDI, hPDI, uPDI and aPDI) and risk of high LDL-C. Red solid line, OR as a function of specific plant-based diet indices adjusted for sex, age, ethnicity, BMI, SES, smoking status, alcohol consumption, physical activity, T2DM, and hypertension; red area,95% CIs. (A) RCS logistic regression models for PDI and ORs of high LDL-C. (B) RCS logistic regression models for hPDI and ORs of high LDL-C. (C) RCS logistic regression models for uPDI and ORs of high LDL-C. (D) RCS logistic regression models for aPDI and ORs of high LDL-C.

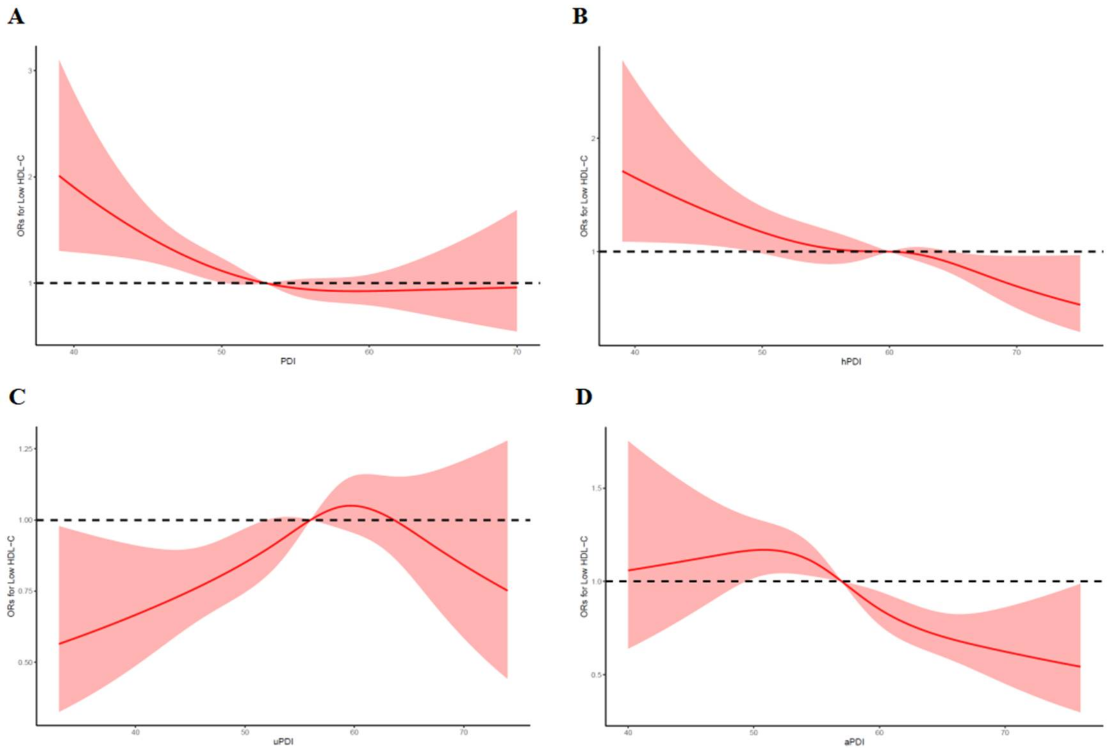

Figure S5. Representation of restricted cubic spline logistic regression models for four plant-based diet indices (PDI, hPDI, uPDI and aPDI) and risk of low HDL-C. Red Solid line, OR as a function of specific plant-based diet indices adjusted for sex, age, ethnicity, BMI, SES, smoking status, alcohol consumption, physical activity, T2DM, and hypertension; red area,95% CIs. (A) RCS logistic regression models for PDI and ORs of low HDL-C. (B) RCS logistic regression models for hPDI and ORs of low HDL-C. (C) RCS logistic regression models for uPDI and ORs of low HDL-C. (D) RCS logistic regression models for aPDI and ORs of low HDL-C.
